# Interleukin-6 inhibition of hERG underlies risk for acquired long QT in cardiac and systemic inflammation

**DOI:** 10.1371/journal.pone.0208321

**Published:** 2018-12-06

**Authors:** Ademuyiwa S. Aromolaran, Ujala Srivastava, Alessandra Alí, Mohamed Chahine, Deana Lazaro, Nabil El-Sherif, Pier Leopoldo Capecchi, Franco Laghi-Pasini, Pietro Enea Lazzerini, Mohamed Boutjdir

**Affiliations:** 1 Cardiovascular Research Program, VA New York Harbor Healthcare System, Brooklyn, New York, United States of America; 2 Department of Cell Biology and Pharmacology, State University of New York Downstate Medical Center, Brooklyn, New York, United States of America; 3 Department of Molecular and Developmental Medicine, University of Siena, Siena, Italy; 4 Centre de Recherche, Institut Universitaire en Santé Mentale de Québec, Department of Medicine, Université Laval, Quebec City, Quebec, Canada; 5 Department of Medical Sciences, Surgery and Neurosciences, University of Siena, Siena, Italy; 6 Departments of Medicine, State University of New York Downstate Medical Center, Brooklyn, New York, United States of America; 7 Department of Medicine, New York University School of Medicine, New York, United States of America; Indiana University School of Medicine, UNITED STATES

## Abstract

Increased proinflammatory interleukin-6 (IL-6) levels are associated with acquired long QT-syndrome (LQTS) in patients with systemic inflammation, leading to higher risks for life-threatening polymorphic ventricular tachycardia such as *Torsades de Pointes*. However, the functional and molecular mechanisms of this association are not known. In most cases of acquired LQTS, the target ion channel is the human ether-á-go-go-related gene (hERG) encoding the rapid component of the delayed rectifier *K* current, *I*_*Kr*_, which plays a critical role in cardiac repolarization. Here, we tested the hypothesis that IL-6 may cause QT prolongation by suppressing *I*_*Kr*_. Electrophysiological and biochemical assays were used to assess the impact of IL-6 on the functional expression of *I*_*Kr*_ in HEK293 cells and adult guinea-pig ventricular myocytes (AGPVM). In HEK293 cells, IL-6 alone or in combination with the soluble IL-6 receptor (IL-6R), produced a significant depression of *I*_*Kr*_ peak and tail current densities. Block of IL-6R or Janus kinase (JAK) reversed the inhibitory effects of IL-6 on *I*_*Kr*_. In AGPVM, IL-6 prolonged action potential duration (APD) which was further prolonged in the presence of IL-6R. Similar to heterologous cells, IL-6 reduced endogenous guinea pig ERG channel mRNA and protein expression. The data *are first* to demonstrate that IL-6 inhibition of *I*_*Kr*_ and the resulting prolongation of APD is mediated via IL-6R and JAK pathway activation and forms the basis for the observed clinical QT interval prolongation. These novel findings may guide the development of targeted anti-arrhythmic therapeutic interventions in patients with LQTS and inflammatory disorders.

## Introduction

IL-6 is a pleiotropic cytokine involved in a variety of biological effects including cardiomyocyte response to injury[1]. IL-6 effects occur either through a membrane-bound receptor, the IL-6R α subunit (classical signaling) or a soluble receptor (sIL-6R) [2] complexed with the signal transduction protein glycoprotein 130 (gp130) leading to the activation of the JAK-related signaling pathways [3, 4]. Previous studies demonstrated that circulating IL-6 levels are elevated in patients with autoimmune-inflammatory disorders and may underlie increased vulnerability to QT interval prolongation that contributes prominently to arrhythmic events and Torsade de Pointes (*TdP)* [5–7].

Cardiac and systemic inflammation are associated with the prolongation of corrected QT (QT_c_) and higher propensity to develop *TdP*, as demonstrated by accumulating data obtained from patients with myocarditis/endocarditis [8], and systemic autoimmune diseases, particularly rheumatoid arthritis (RA) [9], and other connective tissue disease (CTD) [7], as well as in apparently healthy subjects from the general population [10].

Among systemic autoimmune diseases, the largest evidence involves RA and other CTDs. In RA, a chronic disease with high-grade inflammatory burden, the risk of cardiac arrest and sudden cardiac death (SCD) is ~2 times higher than in non-RA patients [11, 12]. Furthermore, prolonged QT_c_ is seen in RA patients [13, 14] and has also been associated with disease severity [14, 15], altered levels of inflammatory markers, including IL-6 [6], as well as presenting as an independent predictor of mortality [14, 16, 17]. RA patients treated with the anti-IL-6R blocker tocilizumab, have also been shown to display a rapid and significant QT_c_ shortening, in addition to decreased levels of C-reactive protein (CRP) and TNF-α [9].

Furthermore, a recent study of a large cohort of RA women demonstrated that inflammation, as assessed by circulating IL-6 levels, correlated more strongly with fatal than non-fatal cardiovascular events [18]. In CTDs patients, a high prevalence of QT_c_ prolongation (up to ~30%) has been reported [7], with circulating IL-1β levels independently predicting the presence of a prolonged QT_c_ [19]. Noteworthy, 10 cases of drug-induced *TdP* in systemic lupus erythematosus patients were reported in the literature, and although CRP level was specifically assessed only in two cases, nevertheless it was elevated in both [7]. These observations provide initial clues as to the potential direct electrophysiological effects of IL-6 on ion channels that can alter action potential duration (APD) and QT_c_ interval.

In the heart, the human ether-á-go-go-related gene, hERG (or KCNH2), which encodes the pore-forming subunit of the rapidly activating component of *I*_*Kr*_, is critical for cardiac repolarization [20, 21]. The functional depression of *I*_*Kr*_ by either drugs, genetic defects or inhibiting autoantibodies [22] causes delayed repolarization leading to prolongation of the QT_c_ on the surface electrocardiogram (ECG) [23] predisposing to SCD [24]. Emerging experimental evidence has shown that inflammatory responses mainly via TNF-α, IL-1β, and IL-6 regulate cardiomyocyte electrophysiological properties [25–29]. TNF-α has been shown to decrease *I*_*Kr*_ and the transient outward current (*I*_*to*_) that is also inhibited by IL-1β [30], while IL-1β [28] and IL-6 [31] increase L-type Ca current (*I*_*Ca*,*L*_) altogether leading to prolongation of APD [28]. However, it is unknown whether IL-6 affects cardiac *I*_*Kr*_.

Recently, we showed increased circulating levels of IL-6 in both RA patients and unselected general population with *TdP* [5], with no measurable changes in TNF-α or IL-1. This outcome led us to hypothesize that selective pathological increases in IL-6 may affect hERG/*I*_*Kr*_, thereby contributing to arrhythmias associated with autoimmune-inflammatory responses [8]. Here, we unravel a novel autoimmune/inflammatory channelopathy as a risk factor for SCD whereby IL-6-mediated inhibition of *I*_*Kr*_ underlie QT_c_ prolongation seen in these patients.

## Methods

### HEK293 cells stably expressing hERG channel

The stably transfected HEK293 cells with hERG channel (HEK-hERG) were a kind gift from Dr Gail Robertson from the University of Wisconsin Madison. They were cultured in Dulbecco’s minimum essential medium (DMEM) supplemented with 10% fetal bovine serum and 100 μg/ml geneticin (G418, Gibco; Grand Island, NY, USA). Cells were washed twice with standard DMEM medium and stored in this medium at room temperature for later use. A coverslip with adherent HEK-hERG cells was placed on the glass bottom of a recording chamber (0.8–1 ml in volume) mounted on the stage of an inverted microscope (Diaphot, Nikon). The internal solution contained (in mmol/L): 130 KCl, 1 MgCl_2_, 0.4 GTP, 5 EGTA, 5 K_2_ATP, and 10 HEPES (pH 7.2). External solution contained (in mmol/L): 137 NaCl, 4 KCl, 1.8 CaCl_2_, 1 MgCl_2_, 10 Glucose, and 10 HEPES (pH 7.4). When filled with internal solution, the pipette resistance was typically 1.5–2 MOhm. Series resistance was compensated 80–90% before each recording. Membrane potentials were corrected for liquid junctional potential. Population current-voltage (*I*–*V)* curves were generated by step depolarizations (-60 to +60 mV), from a holding potential of -80 mV in 10 mV increments for 1 s, followed by a repolarizing step to -40 mV for 5 s to obtain tail currents (*I*_*tail*_). The voltage dependence of steady-state activation of *I*_*Kr*_ in HEK-hERG cells was measured from tail currents recorded at -40 mV following 4 sec steps to voltages in the range -60 to +60 mV and the data fitted using a Boltzmann function to obtain the midpoint of the steady-state activation curve (*V*_1/2_). Currents were sampled at 20 kHz and filtered at 5 or 10 kHz. Traces were acquired at a repetition interval of 10 s. Here and elsewhere cells were pre-treated with IL-6 (20 ng/ml) alone or IL-6 (20 ng/ml) + IL-6R (25 ng/ml) for 40 minutes before experimentation [31]. For the dose-response experiments, in addition to 20 ng/ml, doses of 4 ng/ml and 80 ng/ml of IL-6 were studied in the presence of a constant dose of IL-6R of 25ng/ml. IL-6R is used because the soluble form of the IL-6R (sIL-6R) has been found in body fluids such as blood and high levels of sIL-6R have been reported in several chronic inflammatory and autoimmune diseases hence the ability to regulate cells lacking IL-6R [2]. All experiments were performed at room temperature.

### Guinea-pig ventricular myocytes

Primary guinea pigs’ ventricular cardiac myocytes (used in electrophysiological and biochemical assays) were isolated as previously described [20, 32–34]. Briefly, adult male and female Hartley guinea pigs were deeply anesthetized with isoflurane in accordance with the IACUC approval of this study at the VA New York Harbor Healthcare System and conforming to the NIH guidelines. Hearts were excised, Langendorff perfused with Tyrode solution containing (in mM): 118 NaCl, 4.8 KCl, 1 CaCl_2_, 10 Glucose, 1.25 MgSO_4_, 1.25 K_2_HPO_4_ (pH = 7.4) for 5 minutes. The heart was then perfused with Ca^2+^-free Tyrode solution for 10 minutes before switching to Ca^2+^-free Tyrode solution containing Collagenase B (final concentration, 0.6 mg/ml; Boehringer Mannheim, Indianapolis, IN) for an additional 6 minutes. The heart was subsequently perfused with high-K solution containing (in mM): 70 KOH, 50 L-glutamic acid (potassium salt), 40 KCl, 10 Taurine, 2 MgCl_2_, 10 Glucose, 10 HEPES, 5 EGTA, and 1% albumin (pH 7.4, with KOH) for 5–10 minutes. The digested heart tissue was placed in fresh high-*K* solution, minced into smaller pieces and triturated several times to dissociate the cells. The cell suspension was filtered through a mesh and allowed to settle for 15–20 min. The pellet was resuspended in 10% M199 media and plated on laminin-coated coverslips. Cells were patched 6–8 hours after plating. The external solution used for *I*_*Kr*_ and *I*_*K1*_ recordings contained (in mM): 145 NaCl, 4.5 KCl, 1 MgCl_2_, 1.8 CaCl_2_, 10 HEPES, and 10 glucose (pH 7.4). Ca currents were blocked by the addition of 5 μM nifedipine in the bath solution and the slow delayed rectifier K current (*I*_*Ks*_) was blocked with 100 μM chromanol. The pipette solutions for recording *I*_*Kr*_ and *I*_*K1*_ contained (in mM): 140 KCl, 10 HEPES, 11 EGTA, 1 MgCl_2_, 1 CaCl_2_, 5 MgATP, and 5 K_2_ATP; the pH adjusted to 7.2 with KOH. Currents were recorded in the whole-cell, voltage clamp configuration of the patch-clamp technique using an Axopatch-200B amplifier (Axon Instruments, Inc., Burlingame, CA). *I*_*Kr*_ was recorded using a short 200 ms depolarizing pulse from a holding potential (HP) of -50 mV and test pulses were applied at various voltages from -40 to +80 mV in a 10 mV increment before returning to -40 mV for tail current recording. *I*_*K1*_ was activated from -80 mV to test potential ranging from -120 mV to +10 mV in 10 mV steps for 200 ms. The external solution for *I*_*Na*_ recordings contained (in mM): 20 NaCl, 5 CsCl, 115 tetraethylammonium chloride (TEACl), 1 MgCl_2_, 10 HEPES, and 10 glucose (pH 7.4, with CsOH). L-type Ca current and T-type Ca current were blocked by CoCl_2_ (5 mM) and NiCl_2_ (1 mM), respectively. The internal solution contained (in mM): 140 CsCl, 10 NaCl, 3 MgCl_2_, 5 EGTA, 10 HEPES, and 2 MgATP (pH 7.2, CsOH). *I*_*Na*_ was evoked from -80 mV to test potentials ranging from -70 mV to +20 mV in 10 ms steps for 30 ms. Action potentials were recorded from single ventricular myocytes in current-clamp mode by passing depolarizing currents at subthreshold (1.4 X) intensity. Data were sampled with an A/D converter (Digital 1320A, Axon Instruments) and stored on the hard disk of a computer for subsequent analysis. Currents were sampled at 20 kHz and filtered at 5 or 10 kHz. Traces were acquired at a repetition interval of 10 s.

### Biochemical assays

#### Western blots analysis

Whole cell lysates from HEK293 stably expressing hERG channels and freshly isolated guinea pig cardiomyocytes were used for Western blot analysis. The cells were collected by centrifugation for 5 min at 1,000 g and the pellet was lysed using radioimmunoprecipitation assay (RIPA) buffer (Thermo Fisher Scientific, Walthman, MA) and 10% protease inhibitor cocktail (Sigma, St Louis, MO), incubated on ice for 30 minutes and then centrifuged at 15,000 g for 20 min. The supernatant was collected and the proteins concentration was measured with a Bradford protein assay using EnsSpire 2300 Multimode Plate Reader (PerkinElmer, Waltham, MA). Proteins (50 μg/lane HEK-hERG or 100 μg/lane myocytes) were separated on 4–15% Tris-HCl gel (Bio-Rad Laboratories, Hercules, CA) and electroblotted for 2hr onto polyvinylidene difluoride (PVDF) membrane (Biorad Laboratories, Hercules, CA). Non-specific interactions were blocked using 2.5% non-fat milk (Bio-Rad Laboratories, Hercules, CA), 2.5% BSA (Sigma, St Louis, MO) and 0.1% Tween 20 in Tris-Buffered saline. The membrane was then immunoblotted with anti-K_V_11.1/hERG (extracellular) antibody (1:150; Alomone, Jerusalem, Israel) or rabbit anti-GAPDH antibody (1:1000; Sigma,St Louis, MO) overnight at 4°C. GAPDH expression was used as loading control. The membrane was then probed with anti-rabbit IgG HRP-linked secondary antibody (1:5000; Santa Cruz Biotechnology, Dallas, TX) for 1hr at room temperature and signals were visualized with a chemiluminescence kit (Bio-Rad Laboratories, Hercules, CA). Blots were scanned in a C-Digit blot scanner (LI-COR, Lincoln, NE) at high sensitivity to obtain the image. For quantification of Western blot data, the band intensities of proteins of interest were normalized to their respective GAPDH intensities.

#### Isolation of RNA, cDNA synthesis and RT-PCR

Total RNA was purified using RNeasy fibrous tissue mini-kit (Qiagen, Hilden, Germany). Qubit 2.0 Fluorometer (Thermo Fisher Scientific, Waltham, MA) was used to quantify RNA and to determine the purity of samples. 1 μg RNA was reverse transcribed using High Capacity cDNA Reverse Transcription kit (Applied Biosystems, Waltham, MA) and qPCR was then carried out on cDNA using TaqMan Fast Advanced Mastermix (Applied Biosystems, Waltham, MA). Genes coding for KCNH_2_ (K_v_11.1) and GAPDH were amplified on Applied Biosystem's 7500 Real-Time PCR system. Taqman Gene Expression Assay primers were used, and these were obtained from IDT (Integrated DNA Technologies, Coralville, IA). Primers contained the double quenched probe (5′FAM/ZEN/3′IBFQ) and ROX passive reference dye was used. Gene expression in treated guinea pig cardiomyocytes was represented as a fold change relative to expression in untreated cells.

#### Data and statistical analyses

Electrophysiological data were analyzed off-line using built in functions in clampfit (pCLAMP 10), and Origin software. Quantification of Western blot data was performed by analyzing band intensities of proteins and then normalized to their respective GAPDH intensities. For all electrophysiology and biochemistry assays, the effect of IL-6 alone or IL-6+IL-6R basal *I*_*Kr*_ or hERG expression (mRNA and protein expression) were compared using one-way ANOVA with Bonferroni post-hoc analysis or two-tailed unpaired *t* test for comparisons between groups and considered significant at P < 0.05. Data are reported as means ± S.E.M.

## Results

### IL-6 inhibits *I*_*Kr*_ in HEK293 cells

**Fig 1:**
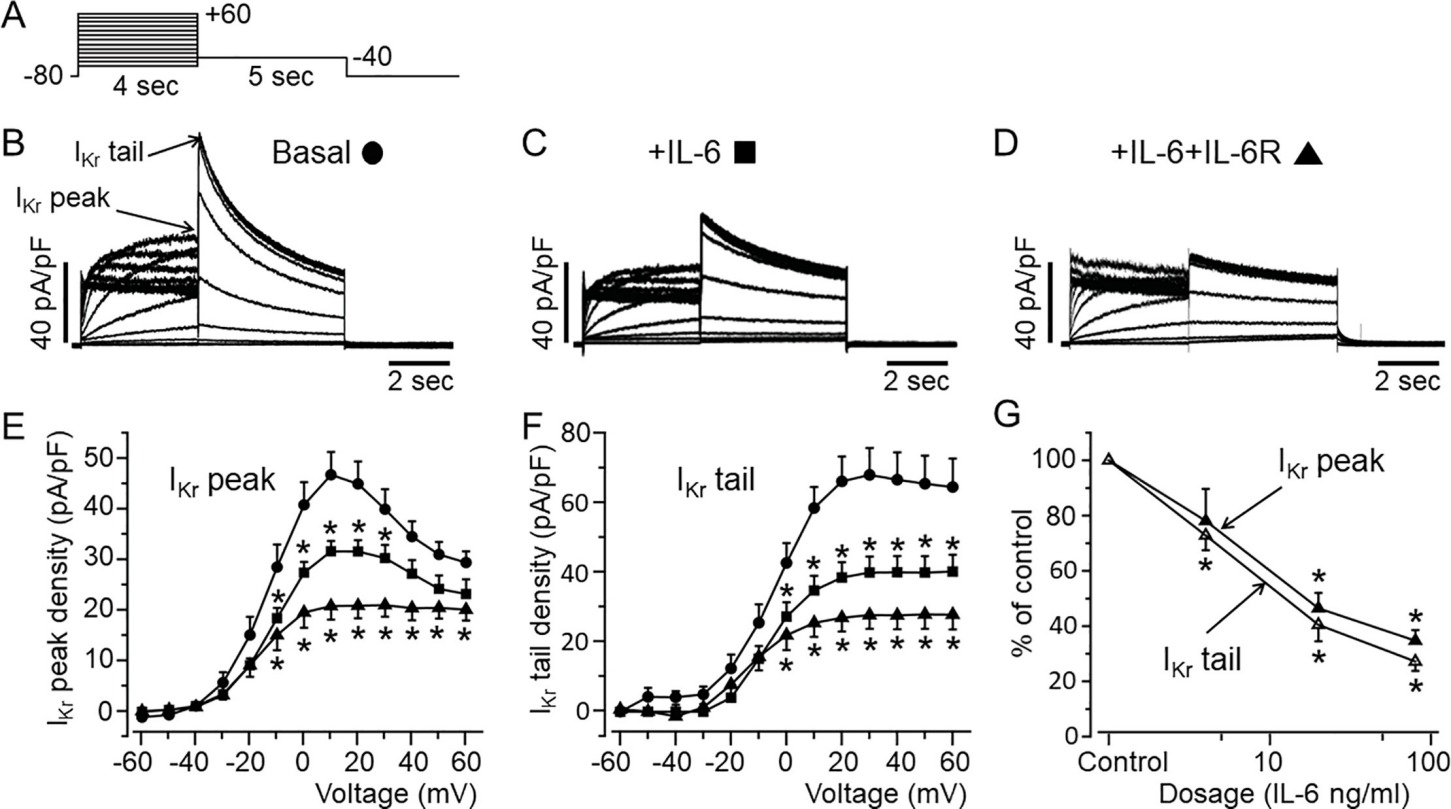
Functional effect of IL-6 on *I*_*Kr*_ in HEK-hERG cells. *A*, Voltage-clamp protocol used to elicit *I*_*Kr*_ in HEK-hERG cells stable expressing hERG channel. *B*, Control hERG current. *Arrows* indicate hERG peak and tail currents. *C*, *I*_*Kr*_ currents measured in cells pre-treated with IL-6 alone or *D*, with IL-6+IL-6R. *E*, *I*_*Kr*_ peak and *F*, tail density-voltage*-* curves for control (circle, n = 21), IL-6 alone (square, n = 18) and IL-6+IL-6R (triangle, n = 9). *G*, The dose-response effects of IL-6+IL-6R on *I*_*Kr*_ peak and tail densities. Data were normalized to control and expressed as percentage of control.

First, we assessed the effects of IL-6 on *I*_*Kr*_ current in HEK-hERG cells using the whole-cell patch clamp technique and the protocol shown in *Fig 1A*. Cells were exposed to IL-6 (20 ng/ml) alone or IL-6 (20ng/ml)+IL-6R (25 ng/ml) at concentrations previously shown to modulate *I*_*Ca*,*L*_ in myocytes after 40 minutes pre-incubation [31]. Compared to basal *I*_*Kr*_ (*Fig 1B*), cells pre-incubated with IL-6 alone (*Fig 1C*) displayed depressed *I*_*Kr*_ densities (*Fig 1E & 1F*). At +20 mV, *I*_*Kr*_ peak densities were inhibited by 29.6% (from 44.9±4.43 pA/pF, n = 21, to 31.6±2.04 pA/pF, n = 18, *P<0.05, *Fig 1E*), and *I*_*Kr*_ tail densities were inhibited by 41.9% at +20 mV (from 66±7.11 pA/pF, n = 21, to 38.3±4.38 pA/pF, n = 18, *P<0.05, *Fig 1F*). In the presence of IL-6R (*Fig 1D*), IL-6 inhibited *I*_*Kr*_ peak densities by 53.6% at +20 mV (from 44.9±4.43 pA/pF, n = 21, to 20.8±2.50 pA/pF, n = 9, *P<0.05, *Fig 1E*) and *I*_*Kr*_ tail densities by 59.5% at +20 mV (from 66±7.11 pA/pF, n = 21, to 26.7±3.94 pA/pF, n = 9, *P<0.05, *Fig 1F*).

Furthermore, we investigated whether the effect of IL-6 on *I*_*Kr*_ is concentration-dependent. At a fixed concentration of IL-6R (25 ng/ml), we assessed the effects of IL-6 at 4 ng/ml and 80 ng/ml of IL-6 on I_Kr_ (*Fig 1G*). At +20 mV, 4 ng/ml of IL-6 reduced *I*_*Kr*_ peak densities by 21.9% (from 44.9±4.43 pA/pF to 35.08±5.71 pA/pF, n = 5, P>0.05, *Fig 1G*) and reduced *I*_*Kr*_ tail densities by 27.2% (from 66±7.11 pA/pF to 48.04±3.52 pA/pF, n = 5, *P<0.05, *Fig 1G*). Increasing IL-6 concentration to 80 ng/ml had a more pronounced effect such that at +20 mV, *I*_*Kr*_ peak densities were reduced by 65.3% (from 44.9±4.43 pA/pF to 15.Y±1.74 pA/pF, n = 13, *P<0.05, *Fig 1G*) and *I*_*Kr*_ tail densities were reduced by 72.8% (from 66±7.11 pA/pF, to 17.93±2.43 pA/pF, n = 13, *P<0.05, *Fig 1G*). Taken together our data demonstrate that IL-6 inhibition of *I*_*Kr*_ is concentration dependent.

**Fig 2:**
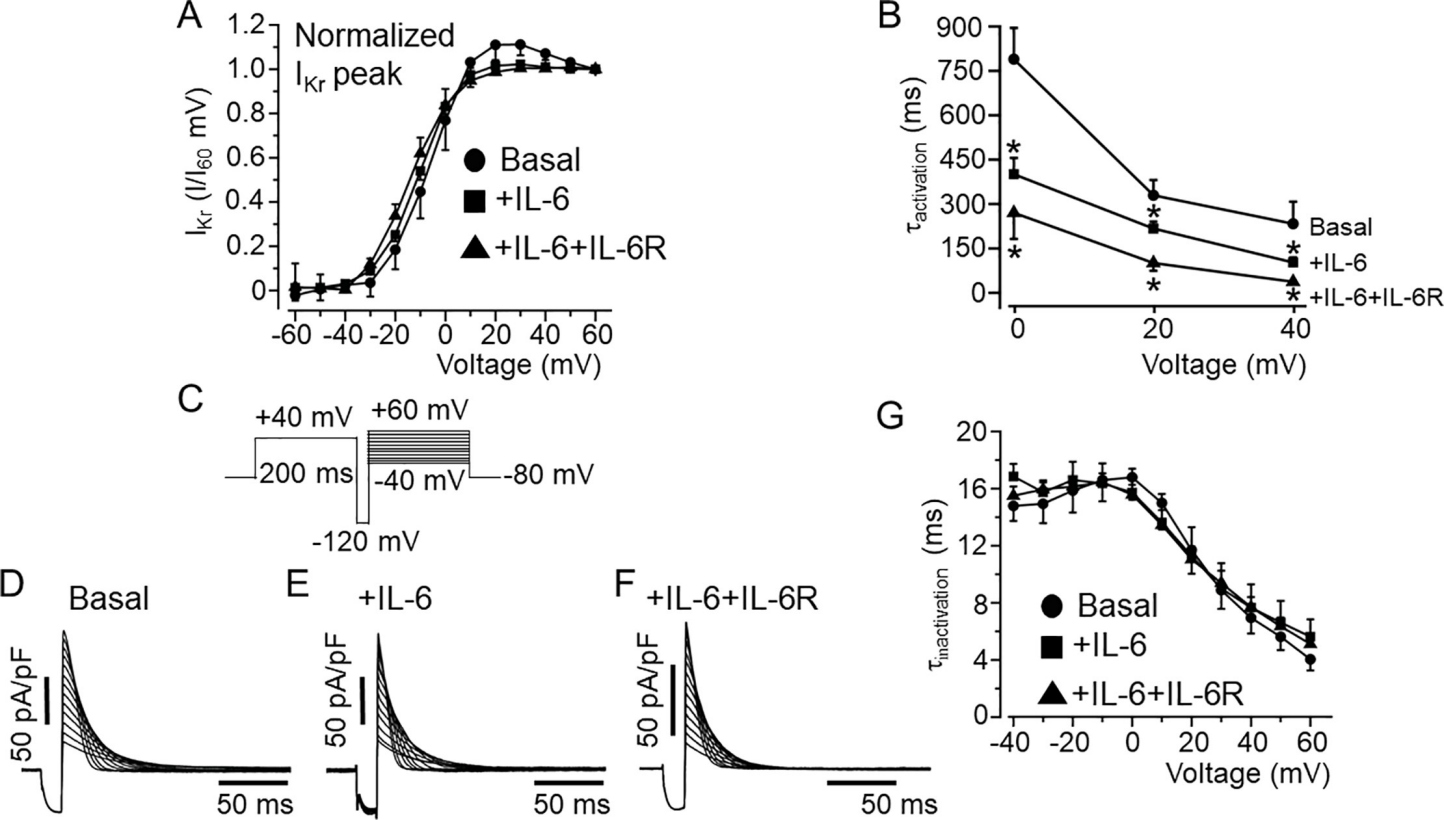
Effects of IL-6 on *I*_*Kr*_ activation and inactivation kinetics in HEK-hERG cells. *A*, Plots of *I*_*Kr*_ tail I/I_+60mV_ for control and in the presence of IL-6 alone and IL-6+IL-6R. Compared to control *I*_*Kr*_ currents (circle, n = 21), the normalized tail currents show that *I*_*Kr*_ activates at more negative potentials in the presence IL-6 (square, n = 18) and IL-6+IL-6R (triangle, n = 9). *B*, Plots of activation time course (τ_activation_) and voltage relationship for basal *I*_*Kr*_ (circle, n = 21), and in the presence of IL-6 (square, n = 18) or IL-6+IL-6R (triangle, n = 9). The time course of inactivation (τ_inactivation_) was examined using the protocol shown in *C*. *D*, representative *I*_*Kr*_ current traces recorded under basal conditions, and in the presence of IL-6 (*E*) or IL-6+IL-6R (*F*). *G*, graph of the voltage-dependence of the τ_inactivation_ during basal *I*_*Kr*_ (circle, n = 8), +IL-6 (square, n = 7), +IL-6+IL-6R (triangle, n = 5) conditions.

We next examined the effect of IL-6 and IL-6+IL-6R on the biophysical properties of *I*_*Kr*_. First, we analyzed and compared the *I-V* relationships for activation measured during the depolarizing steps. *I*_*Kr*_ tail currents were normalized to the maximum current at +60 mV and plotted as a function of voltage (mV) and fitted to a Boltzmann equation to obtain activation curves (*Fig 2A*). IL-6 alone (*Fig 2A*), produced a small leftward shift in the midpoint of the steady-state activation (or *V*_*1/2*_) from -5.86±2.45 mV (n = 21) to -10.75±1.32 mV (n = 18, P>0.05), but produced a significant shift to -14.6±2.11 mV (n = 9, *P<0.05, *Fig 2A*), in the presence of IL-6+IL-6R (*Fig 2A*) respectively.

To determine the *I*_*Kr*_ activation time course (τ_activation_), the data were fitted with a single exponential function. *I*_*Kr*_ measured in HEK-hERG cells pretreated with IL-6 alone or IL-6+IL-6R produced faster activation kinetics (*Fig 2B*). At +20 mV, τ_activation_ accelerated from 329±52 ms (n = 21) to 219±16 ms, (n = 18, *P<0.05) with IL-6 and to 103.2±20.1 ms (n = 9, *P<0.05) in the presence of IL-6+IL-6R demonstrating that IL-6+IL-6R exert a profound effect on the activation kinetics of *I*_*Kr*_. The next series of experiments examined the effects of IL-6 on the inactivation kinetics of *I*_*Kr*_. A three-step protocol (*Fig 2C*) to isolate inactivating currents and determine the kinetics of inactivation was used [35–37]. hERG channels were activated by a 200 ms depolarizing step to +40 mV followed by a brief hyperpolarizing step to -120 mV for 10 ms to allow the channels to recover from inactivation. The hyperpolarizing step was followed by a step to various test potential from -40 mV to +60 mV. The estimated time constant of inactivation (τ_inactivation_) was measured by fitting the current traces to a single exponential. As illustrated in *Fig 2*, there was no significant difference in current traces and τ_inactivation_ values obtained for basal *I*_*Kr*_ (*Fig 2D*), and currents measured in the presence of IL-6 alone (*Fig 2E*), or IL-6+IL-6R (*Fig 2F*) within the voltage range studied (*Fig 2G*).

**Fig 3:**
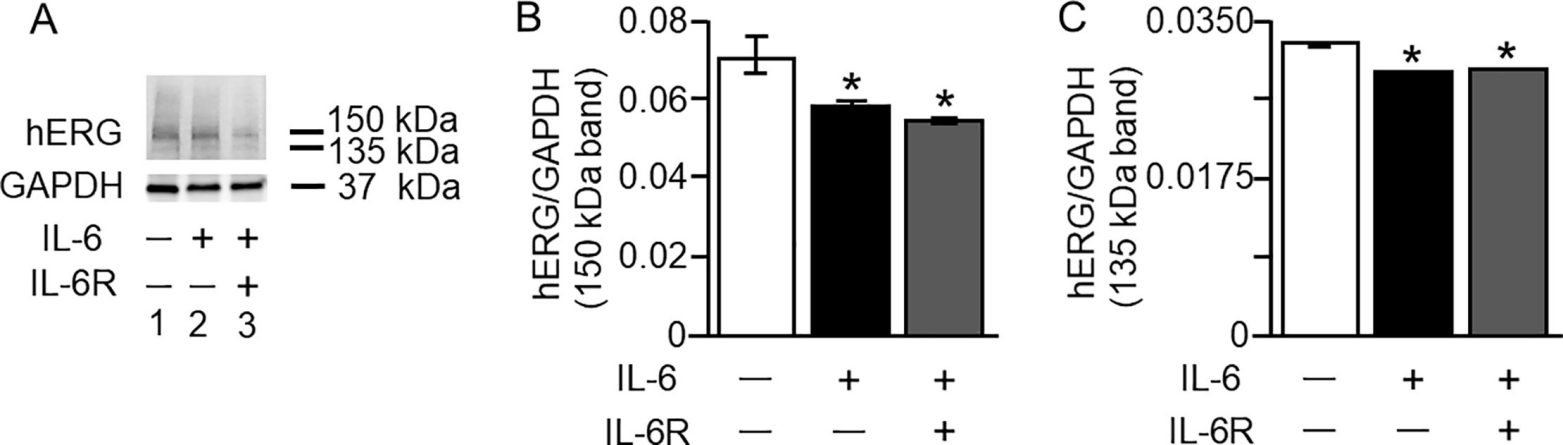
Effect of IL-6 on *I*_*Kr*_ protein expression in HEK-hERG cells. Western blot analysis of the *I*_*Kr*_ channel hERG expression in HEK-hERG cells from control cells and in cells pre-treated for 40 min with IL-6 (20 ng/ml) alone or IL-6 (20 ng/ml)+IL-6R (25 ng/ml). *A*, compared to control hERG protein expression (*Lane 1*), exposure to IL-6 (20 ng/ml) reduced the expression of the 150 kDa and 135 kDa (*Lane 2*) hERG proteins. A further decrease in the bands was observed (*Lane 3*) with pretreatment with IL-6 (20 ng/ml) + IL-6R (25 ng/ml). Compared to untreated HEK-hERG cells (*Open bar*, *B and C*), normalized values of 150 kDa (*B*) and 135 kDa (*C*) hERG bands/GAPDH revealed significant down-regulation of the protein expression of hERG in the presence of IL-6 alone (*Black Bars*, *B and C*) or IL-6+IL-6R (*Grey Bars*, *B and C*). Equal amounts of protein were loaded in each lane. The 37 kDa bands represent GAPDH.

Next, we assessed whether IL-6 affected hERG expression in HEK-hERG cells. Western blot analysis was used to examine protein expression of hERG channels in control untreated cells and in cells pretreated for 40 minutes with IL-6 alone or IL-6+ILR. As illustrated in *Fig 3A*, (*Lane 1*), untreated control cells displayed bands at 150 kDa and 135 kDa, which are specific for the hERG channel. Cells pretreated with IL-6 (*Fig 3A, Lane 2*), or IL-6+IL-6R (*Fig 3A, Lane 3*), revealed reduced expression of the 150 kDa band. Average densitometry analysis demonstrated that IL-6 significantly reduced the 150 kDa expression by 16% (from 0.070±0.000311 to 0.059±0.000427, n = 5, *P<0.05, *Fig 3B*), and by 21% (from 0.070±0.0001 to 0.055±0.000427, *P<0.05) in the presence of IL-6R. Similarly, the 135 kDa band (*Fig 3A*) was reduced by IL-6 from 0.033±0.0001 to 0.029±0.0000424 (n = 5, *Fig 3C*, *P<0.05) and by IL-6+IL-6R to 0.029±0.000015 (n = 5, *Fig 3C,* *P<0.05).

### IL-6 reduced *I*_*Kr*_ density through the IL-6R and JAK signaling pathways in HEK-hERG cells

**Fig 4:**
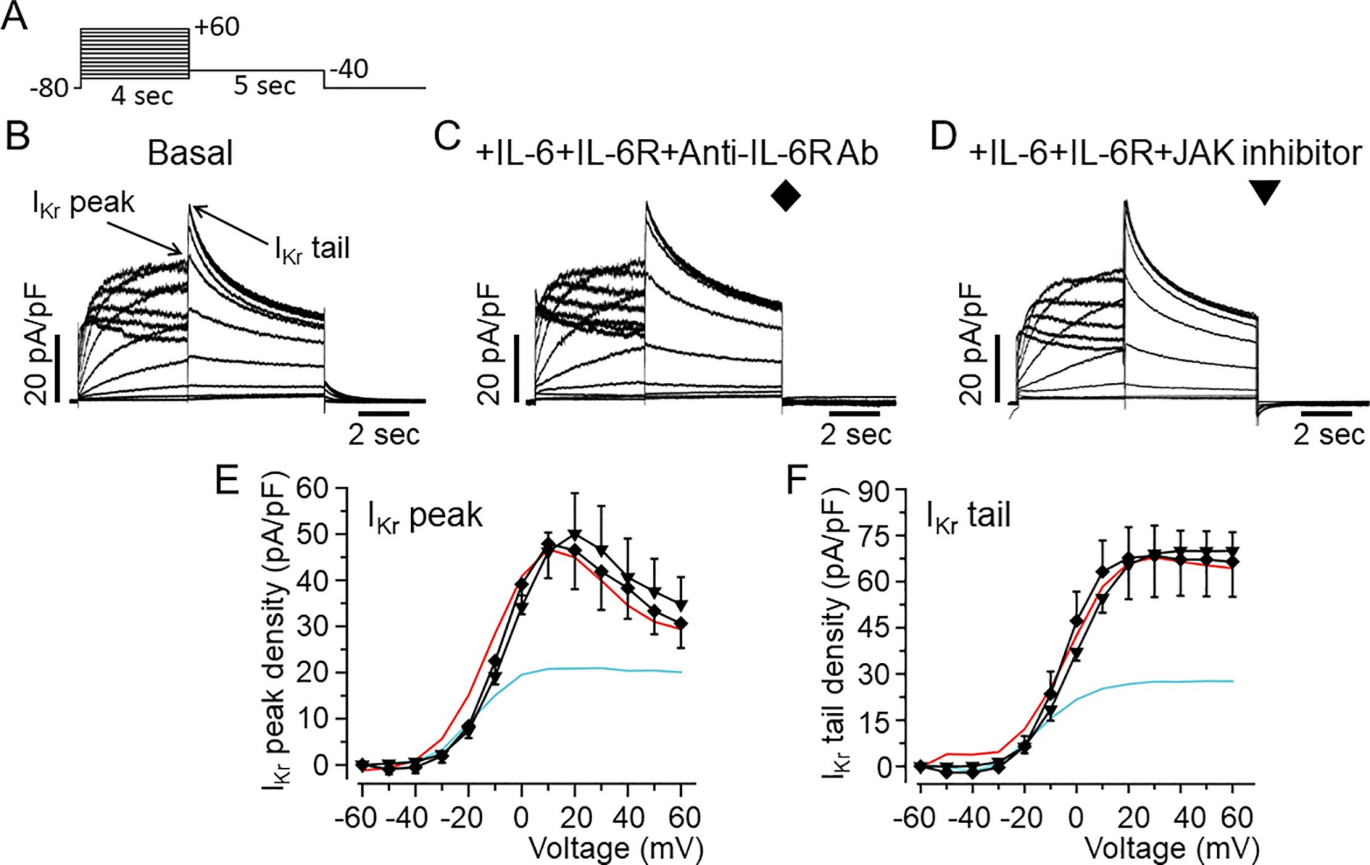
Inhibition of *I*_*Kr*_ by IL-6 is prevented by IL-6R and Janus Kinase blockade in HEK293 cells. *A*, Voltage-clamp protocol used to generate *I*_*Kr*_ in HEK-hERG cells stably expressing hERG channel. *B*, Basal hERG currents. *Arrows* indicate hERG peak and tail currents. *C*, Representative *I*_*Kr*_ traces from HEK-hERG cells pretreated with IL-6+IL-6R in the presence of anti-IL-6R antibody (Ab). *D*, *I*_*Kr*_ currents measured in HEK-hERG cells pre-treated with IL-6+IL-6R and the JAK inhibitor-1. Plots of *I*_*Kr*_ peak current density-voltage (*E*) and tail current density-voltage curves (*F*) in the presence of anti-IL-6R antibody (n = 4) and the JAK inhibitor-1 (n = 4). *I*_*Kr*_ peak current and tail density-voltage curves for *I*_*Kr*_ measured in basal condition (*Red line*) and in cells pre-treated with IL-6+IL-6R (*Cyan line*) are shown for comparison.

The results presented in *Fig 1* show that inhibition of *I*_*Kr*_ by IL-6 is more profound with IL-6R. To investigate whether IL-6 acts on *I*_*Kr*_ via IL-6R in HEK-hERG cells, we incubated IL-6R (25 ng/ml) with an inhibitory mouse monoclonal anti-IL-6R antibody (20–100 μg/ml, H-7: sc-373708, Santa Cruz, Dallas, TX, USA) for 40 minutes at room temperature. The mixture was then added to cells in combination with IL-6 (20 ng/ml) for 40 minutes before measuring *I*_*Kr*_ using the protocol shown in *Fig 4A*. Control *I*_*Kr*_ peak and tail currents measured in untreated HEK-hERG cells is shown in *Fig 4B*. In 4 separate experiments pre-treatment of cells with anti-IL-6R antibody (*Fig 4C*), completely prevented the inhibitory effects of IL-6 on *I*_*Kr*_, but did not alter the currents when used alone (Data not shown). In the presence of anti-IL-6R antibody, *I*_*Kr*_ peak and tail current densities were 46.5±8.44 pA/pF (n = 4, P>0.05, *Fig 4E*) and 67.6±10.02 pA/pF (n = 4, P>0.05, *Fig 4E*) respectively, when compared to basal *I*_*Kr*_. To investigate the involvement of Janus Kinase (JAK), we assessed *I*_*Kr*_ in the presence of IL-6+IL-6R in HEK-hERG cells pre-exposed to a JAK inhibitor-I (5 μM) [38] for 30 minutes. Pre-treatment of cells with JAK inhibitor-I also prevented the inhibitory effect of IL-6 on *I*_*Kr*_ (*Fig 4D)*. With JAK inhibitor-I, averaged *I*_*Kr*_ peak and tail current densities were 50.05±8.81 pA/pF (n = 4, P>0.05, *Fig 4E and 4F*) and 65.46±11.2 pA/pF (n = 4, P>0.05, *Fig 4F*) respectively and similar to control *I*_*Kr*_ peak (*Fig 4E*) and tail (*Fig 4F*) densities. Normalizing peak *I*_*tail*_ curves to the maximal current at +60 mV obtained in the presence of anti-IL-6R antibody and JAK inhibitor-I revealed plots and *V*_*1/2*_ values that were like control (*Fig 4E*). *V*_*1/2*_ was -5.67±1.33 mV (n = 4, P>0.05, and -3.46±4.16 mV, n = 4, P>0.05) in the presence of anti-IL-6R antibody and JAK inhibitor-I respectively.

### IL-6 inhibits *I*_*Kr*_ via Janus Kinase in freshly isolated guinea-pig ventricular cardiomyocytes

**Fig 5:**
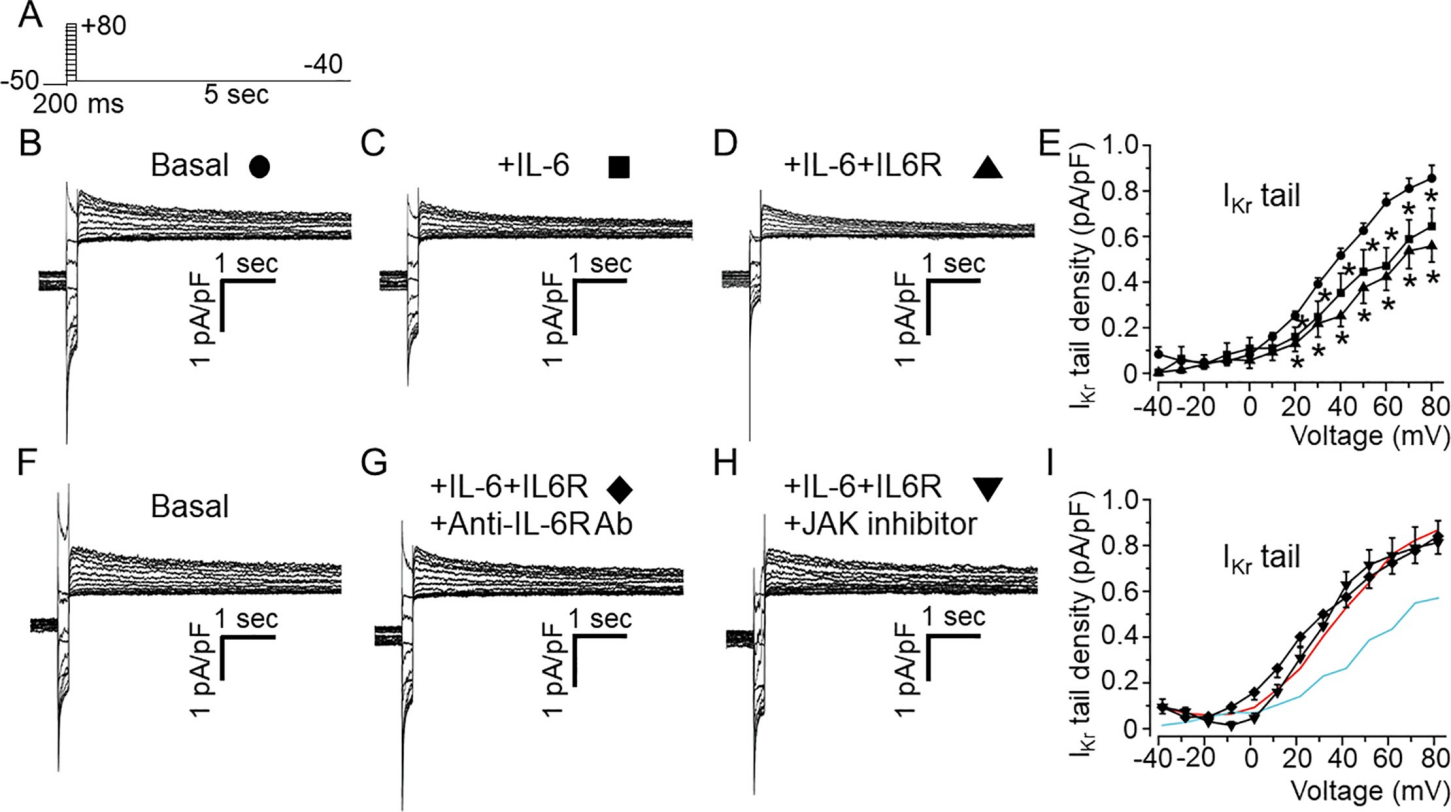
Effects of IL-6 and IL-6+IL-6R on *I*_*Kr*_ in adult guinea-pig ventricular myocytes. *A*, Voltage protocol used for evoking *I*_*Kr*_ in freshly isolated ventricular myocytes from adult guinea-pig heart. Tail current traces measured in the presence of 100 μM chromanol 293B and 5 μM nifedipine in control untreated cardiomyocyte (*B*, circle, n = 15), and myocytes pre-treated with IL-6 alone (*C*, square, n = 8) or IL-6 +IL-6R (*D*, upward triangle, n = 5). *E*, Population *I*_*Kr*_ tail density-voltage curves in basal, IL-6- and IL-6R-treated adult guinea-pig ventricular cardiomyocytes. *F*, Representative *I*_*Kr*_ traces in basal condition, (*G*) pre-treatment with IL-6+IL-6R in the presence of anti-IL-6R antibody (Ab) at 100 μg/ml or, *H*) a JAK inhibitor-I (5 μM). *I*, the mean *I*_*Kr*_ tail density-voltage curves show that the inhibitory effect of IL-6+IL-6R on *I*_*Kr*_ is reversed in the presence of anti-IL-6R antibody (diamond, n = 12) or JAK inhibitor I (downward triangle, n = 5). For visual comparison, data for control *I*_*Kr*_ (*Red trace*) and in the presence of IL-6+IL-6R (*Cyan trace*) in *I*.

We next assessed the effect of IL-6 on native *I*_*Kr*_ from AGPVM. This was investigated by using chromanol 293B (100 μM) to block the slowly-activating delayed rectifier K current, *I*_*Ks*_ in addition to the use of a short-test depolarizing pulse protocol (200 ms, *Fig 5A*) during which a negligible amount of *I*_*Ks*_ will be activated, but allows a sufficient activation of a tail current upon repolarization which is largely due to slow deactivation of *I*_*Kr*_ [39]. In untreated myocytes, short depolarizing pulses evoked large outward deactivating tail currents (*Fig 5B*). Like the observations in HEK-hERG cells, IL-6 alone (*Fig 5C*) or IL-6+IL-6R (*Fig 5D*) significantly reduced currents at potentials positive to 10 mV. Compared to averaged control values (0.24±0.02 pA/pF, n = 15, *Fig 5E*) measured at +20 mV, *I*_*Kr*_ current densities were reduced by 45.8% (or to 0.13±0.02 pA/pF, n = 8 *P<0.05) in the presence of IL-6 alone, and by 58.3%, (0.10±0.05 pA/pF, n = 5, *P<0.05) with IL-6+IL-6R. To test whether IL-6 exerted its inhibitory effect on *I*_*Kr*_ via IL-6R and downstream JAK signaling, experiments were performed in the presence of anti-IL-6R monoclonal antibody (100 μg/ml), and JAK inhibitor-I (5 μM) respectively. Compared to control *I*_*Kr*_ (*Fig 5F*), pretreatment with IL-6+IL-6R+anti-IL-6R antibody (*Fig 5G*) or IL6+IL-6R+JAK inhibitor-I (*Fig 5H*) abolished the inhibitory effects of IL-6+IL-6R (*Fig 5I*).

**Fig 6:**
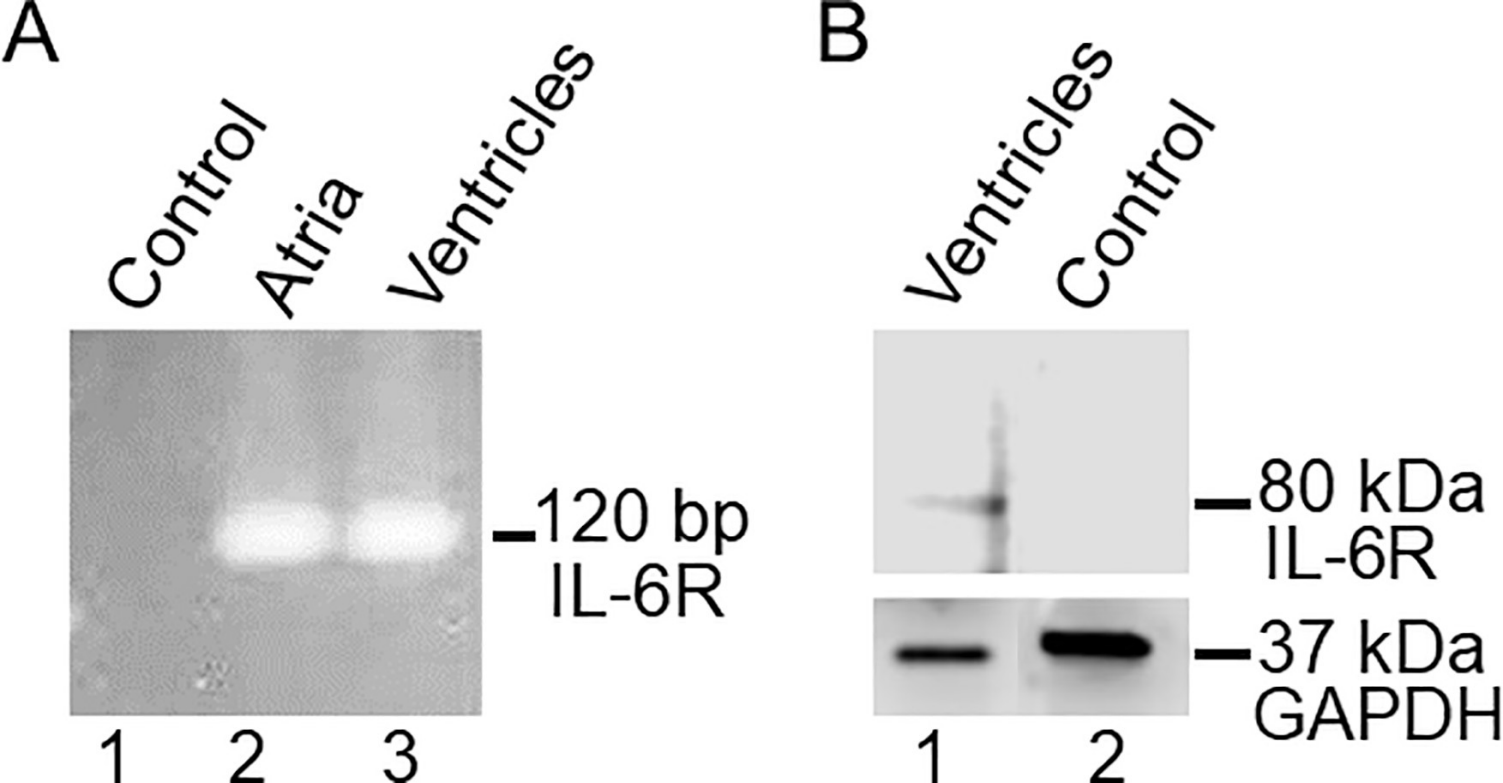
Transcript and protein expression of IL-6R in guinea pig heart. *A*, qRT-PCR assay for detection of IL-6R mRNA expression in guinea-pig heart. Compared to control (*Lane 1*), IL-6R (120 bp) mRNA expression was detected in both atria (*Lane 2*) and ventricles (*Lane 3*) consistent with an endogenous IL-6R expression in guinea pig heart. *B*, Western blot of IL-6R from guinea-pig ventricular cell lysates. The blot was probed with anti-IL6Rα H-7. The band around 80 kDa (*Fig 6B*, *Lane 1*) represents IL-6Rα which was absent when IL-6Rα Ab was pre-incubated with its own blocking peptide (*Fig 6B*, *Lane 2*).

We examined whether the endogenous IL-6Rα transcript and protein are expressed in guinea-pig heart using qRT-PCR and Western blots. Compared to control (water) (*Lane 1*), the results show that IL-6R is robustly expressed as a 120 bp band in both the atria (*Lane 2*) and ventricles (*Lane* 3, *Fig 6A*) in guinea-pig heart. Western blots of guinea pig ventricular cell lysates probed with anti-IL6Rα IL-6 antibody (H-7) further show that IL-6Rα protein expression which is manifested as an 80 kDa band (*Fig 6B, lane 1*) that was not seen when IL-6Rα antibody was pre-incubated with its own blocking peptide (*Fig 6B, lane 2*), in-line with reported data in rat heart [38].

**Fig 7:**
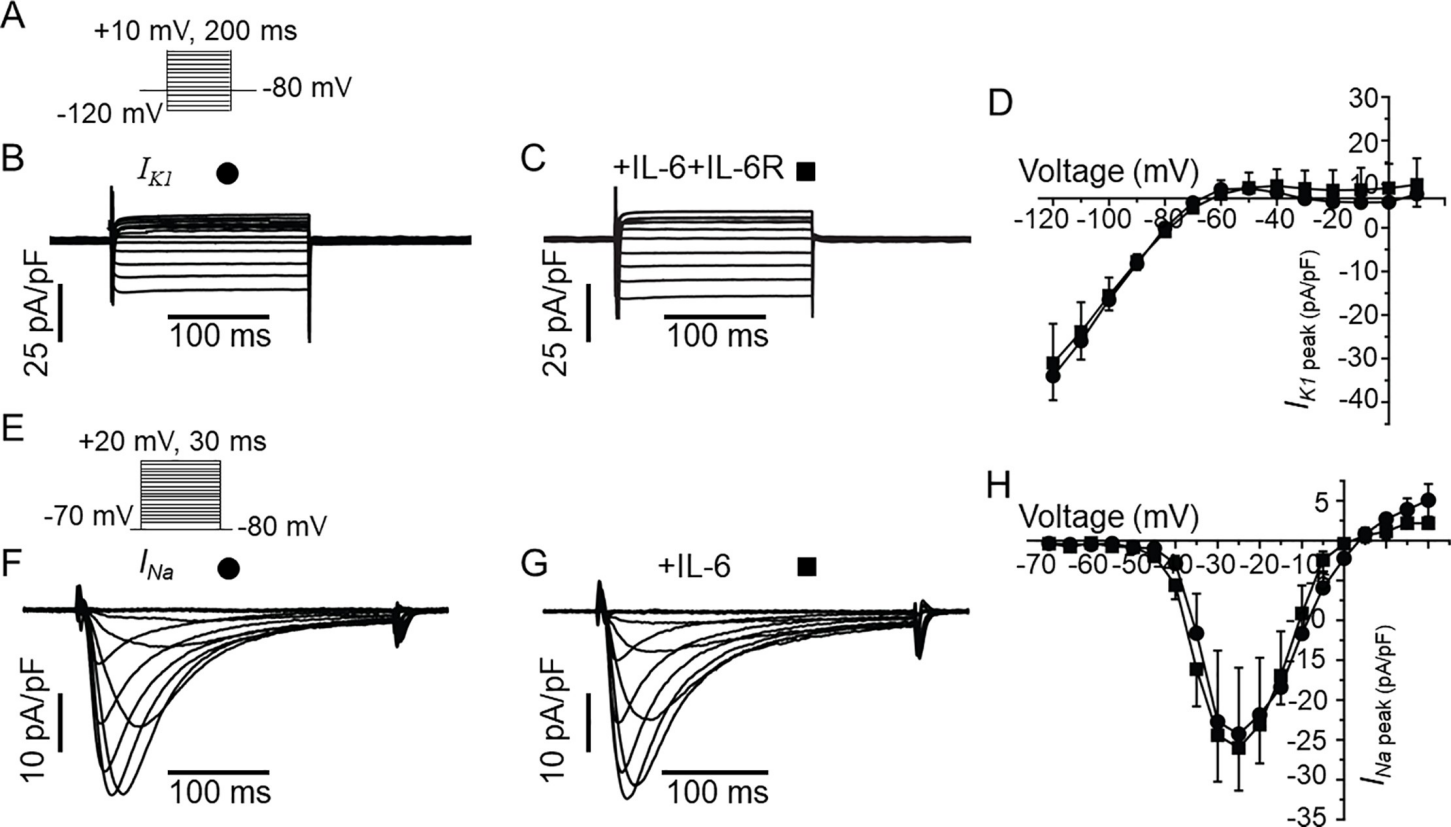
IL-6 has no effect on *I*_*K1*_ or *I*_*Na*_ in guinea-pig ventricular myocytes. *A*, Voltage protocol for activation of *I*_*K1*_. Representative *I*_*K1*_ traces for a control myocyte (*B*) and from an IL-6+IL-6R pre-treated myocyte (*C*). *D*, Population *I*_*K1*_ peak density-voltage curves show that *I*_*K1*_ was not altered in IL-6-IL-6R pre-treated myocytes (square, n = 4) compared to untreated myocytes (circle, n = 3) at all voltages between -120 mV to +10 mV. *E*, The voltage protocol for evoking *I*_*Na*_. Representative traces showing that *I*_*Na*_ measured in control cardiomyocytes (*F*) are identical to *I*_*Na*_ recorded in myocytes pre-treated with IL-6 alone (*G*). *H*, pooled data for *I*_*Na*_ peak-voltage in untreated (circle, n = 6), and IL-6-treated myocytes (square, n = 6).

Furthermore, IL-6 did not have any measurable effects on the densities of the inwardly rectifying *K* current (*I*_*K1*_, *Fig 7C*) and voltage-gated Na current (*I*_*Na*_, *7G*) at all voltages tested (*Fig 7D & 7H*) demonstrating that the inhibition of *I*_*Kr*_ by IL-6 is specific and does not indiscriminately inhibit all currents.

### IL-6 regulates guinea-pig ERG expression in ventricular cardiomyocytes

**Fig 8:**
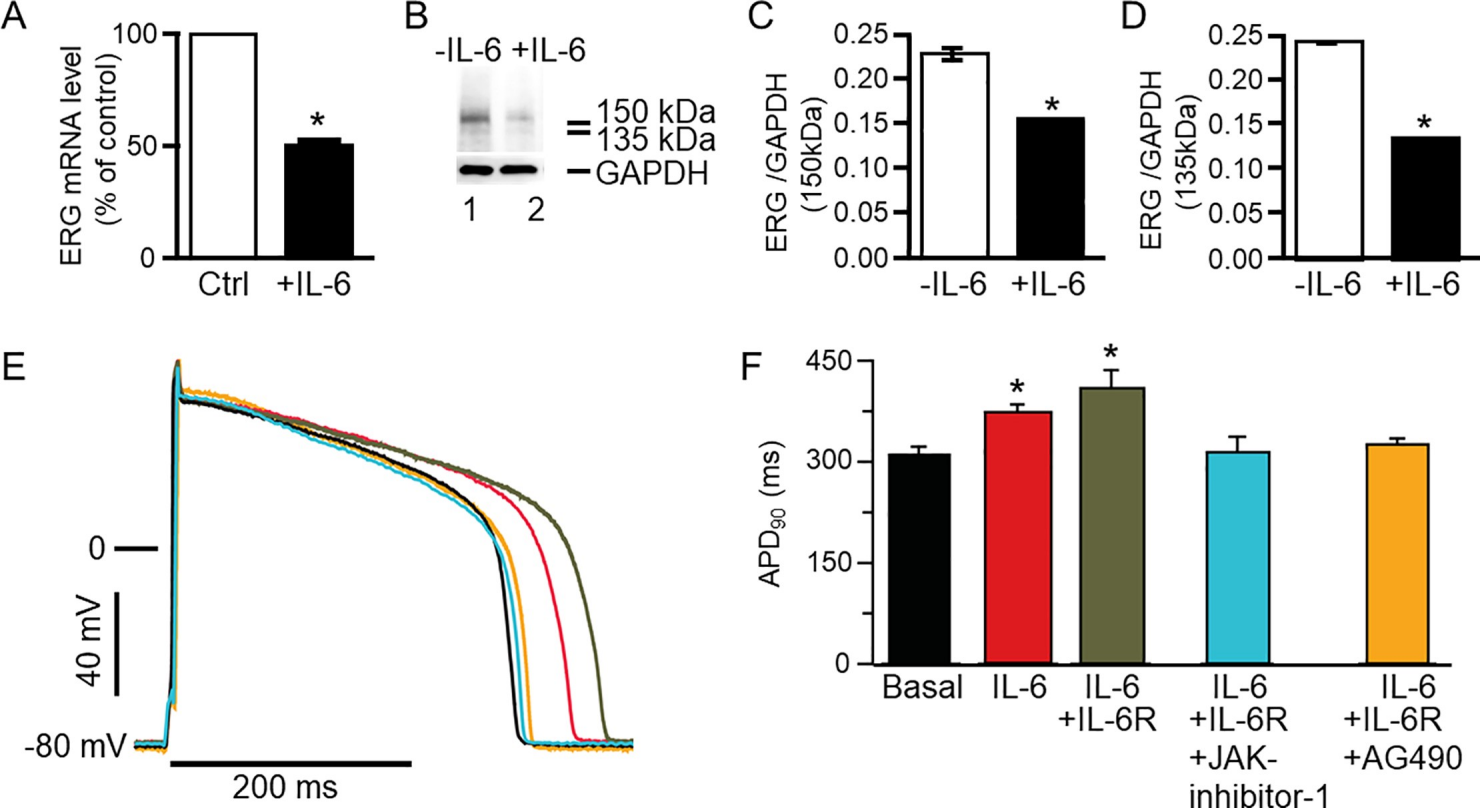
Effect of IL-6 on *guinea pig ERG* expression and action potential in ventricular myocytes. *A*, qRT-PCR was used to determine guinea pig ether-á-go-go-related gene (ERG) mRNA expression in control ventricular myocytes and in myocytes pre-treated with IL-6 (20 ng/ml). *B*, Western blot assay of guinea pig ERG protein expression in control (*Lane 1*) and IL-6 pretreated (for 40 mins) myocytes (*Lane 2*). Values were normalized to the GAPDH signal and expressed as % of control or in the absence of IL-6. Each sample was analyzed in triplicate. 150 kDa and 135 kDa ERG bands were identified in lane 1 but were significantly less dense in lane 2 loaded with IL-6 (20 ng/ml) pretreated ventricular myocytes’ lysate. *C*, Comparison of the relative abundance of the 150 kDa ERG band in untreated and IL-6 treated myocytes. *D*, Comparison of the relative abundance of the 135 kDa ERG band in untreated and IL-treated myocytes. The 37 kDa bands represent GAPDH. *E*, Action potential waveforms recorded in control myocytes (basal conditions without IL-6, *Black trace*) and in the presence of IL-6 alone (*Red trace*), IL-6+IL-6R (*Grey trace*), IL-6+IL-6R+JAK inhibitor-1 (*Cyan trace*) and another JAK inhibitor AG490 (*Orange trace*). *F*, Pretreatment of myocytes with IL-6 and IL-6+IL-6R significantly prolonged APD_90_ and JAK inhibitor I and AG490 completely reversed the prolongation of the action potential.

To determine whether the effects of IL-6 on hERG channel expression observed in HEK-hERG cells are recapitulated in cardiomyocytes, we performed qRT-PCR and Western blot assays. IL-6 (20 ng/ml) significantly reduced transcript (*Fig 8A*) and protein expression of adult guinea pig ERG ventricular myocytes (*Fig 8B–8D*). Relative to control (or in the absence of IL-6), guinea pig ERG mRNA was significantly reduced by 48% (n = 3, *P<0.05). Guinea pig ERG protein expression quantified as the 150 kDa (*Lane 1*, *Fig 8B*) band is significantly reduced by 32% (or from 0.229±0.002, n = 5, to 0.157±0.00018, n = 5, *P<0.05), while the 135 kDa band (*Lane 2*, *Fig 8B*) is significantly reduced from 0.245±0.000447 to 0.138±0.000671 (or by 43.6%, n = 5, *P<0.05, *Fig 8D*).

### Modulation of ventricular action potential duration by IL-6/IL-6R/JAK

Finally, we examined the effect of IL-6/IL-6R on action potential duration measured in AGPVM. IL-6 significantly increased APD at 90% repolarization (APD_90_) from 310±12.4 ms (*Black trace)*, to 371±13.9 ms (*Red trace;* 19%, n = 11, *P<0.05*, Fig 8E and 8F*), but had a more pronounced effect in the presence of IL-6R (*Grey trace*) as APD_90_ increased to 408±27.8 ms (or by 32%, n = 14, *P<0.05). Inhibition of JAK with JAK inhibitor-I (5 μM, *Cyan trace*) [38], or AG490 (5 μM, *Orange trace*) [40], completely prevented APD prolongation (*Fig 8E and 8F*). Compared to control APD_90_ (310±12.4 ms), values were 312.5±24.7 ms (P>0.05, n = 7, *Fig 8E and 8F*) and 325.5±9.43 ms (n = 6, P>0.05, *Fig 8E*) with JAK inhibitor and AG490 respectively.

## Discussion

Autoimmune inflammatory disorders are associated with an increased risk for QT_c_ prolongation and *TdP*, thereby contributing to the incidence of SCD [15, 22]. Here, we show that IL-6 suppresses *I*_*Kr*_ in heterologous cells and myocytes resulting in prolonged APD. Furthermore, IL-6 also markedly blunted hERG/*I*_*Kr*_ channel mRNA and protein expression. To our knowledge, there have been no previous reports on IL-6 modulation of *I*_*Kr*_ in any cell type. Thus elevated IL-6 levels, reduced *I*_*Kr*_ and associated action potential prolongation described here may contribute to delayed repolarization and associated ventricular arrhythmias such as *TdP* reported in patients with autoimmune inflammatory disorders [22].

### Comparison to previous studies on cytokines and cardiac *K* channels

As pointed out above, there are no studies on the functional impact of IL-6 on hERG/*IKr* channel to date. However, and in support to this study, *Wang et al* [25] showed that another cytokine, TNF-α reduced *I*_*HERG*_ in HEK293 cells and *I*_*Kr*_ in dog ventricular myocytes. TNF-α has also been shown to reduce *I*_to_ in rat ventricular myocytes [26, 27]. Similarly, IL-1β inhibited *I*_to_ in mouse ventricular cardiomyocytes [30]. Therefore, taken together our data are consistent with the concept of modulation of cardiac *K* channels by cytokines.

We also found that inhibition of IL-6R or JAK prevented the effects of IL-6 on *I*_*Kr*_ recorded in heterologous cells and myocytes indicating that the inhibitory effect of IL-6 on *I*_*Kr*_ involves IL-6R and gp130 downstream pathways. This is in line with previous reports that JAK is a downstream pathway of IL-6 via gp130 in cardiomyocytes [41]. Other studies demonstrated that excess levels of pro-inflammatory cytokines also play a role in causing LQT, possibly through mechanisms that involve reactive oxygen species [25, 26] and ceramide signaling pathway [42, 43]. Our findings reveal a novel and/or distinct IL-6/IL-6R-JAK-*I*_*Kr*_ signaling pathway involved in acquired LQT in inflammation.

There have also been reports of cytokine’s effects on cardiac *K* channel subunit gene and protein expression with contradicting results. While Petkova-Kirova *et al* [29] and Fernandez-Velasco *et al* [26] reported reduced cardiac *K* channels protein expression with TNF-α, Grandy & Fiset [44] reported no changes. In this study, IL-6 decreased expression of hERG proteins which occurred within 40 minutes suggesting that IL-6 alone or in combination with IL-6R may depress hERG expression by accelerating the channel turnover at multiple levels including at the transcriptional, translational as well as channel turnover at the cell-surface. Furthermore, in addition to significant inhibition in *I*_*Kr*_ current, we find that IL-6 pre-treated HEK-hERG cells displayed reduced I_*Tails*,_ a leftward shift in *V*_*1/2*_ of activation, and time-course of activation both of which were further pronounced in the presence of IL-6R. These mechanistic insights are likely to have important implications for predicting the functional impact of temporal changes in cytokine levels, functional expression of *K* channels, and cardiac repolarization. Therefore, the inhibitory effect of IL-6 on *I*_*Kr*_ can be attributed, at least in part, to both channel gating and protein trafficking.

Previous studies have also shown reduced outward *K* currents and a prolonged APD in isolated myocytes incubated with TNF-α [25, 26] or IL-1β [30]. In contrast, Grandi and Fiset [44] showed that ventricular APD was not altered in TNF-α treated mice, despite reduced *I*_to_ and *I*_Kur_. Recently George *et al* [45] also demonstrated lack of a prolonged APD with TNF-α in atrial myocytes isolated from adult guinea-pigs although voltage-gated *K* currents were not measured in these studies. However, APD prolongation and heart failure were reported by London *et al* [46] in transgenic mice overexpressing TNF-α and by Wang *et al* [25] in dog ventricular myocytes. Therefore, one can speculate that our findings suggest that IL-6 may also be an important contributor to *I*_*Kr*_ reduction in heart failure [47].

Finally, IL-6 is emerging as one of the relevant cytokines involved in the inflammatory process with cardiac electrophysiological consequences [5]. The physiological relevance of this study is underscored by focusing specifically on the individual effects of IL-6 on *I*_*Kr*_ channel function. This is an important step prior to identifying whether common mechanisms underlie the complex electrical remodeling process associated with the cumulative effects of inflammatory factors that occur during the complex process of cardiac and systemic inflammation that predispose to arrhythmic events. IL-6 inhibition has also been developed as a therapy for diseases associated with inflammation [48]. These efforts are likely to increase our understanding of how IL-6 and its inhibitors may affect cardiac electrical function. In this regard, the IL-6R inhibitor tocilizumab [49], which is widely used clinically to treat RA [50], may also have an emerging role as an anti-arrhythmic drug [9, 51].

## Conclusion

Overall, the present study is first to demonstrate that pathologically elevated IL-6 levels can negatively modulate *I*_*Kr*_. Our findings are consistent with the notion that changes in IL-6 in individuals with systemic inflammation related to autoimmune disorders (but also possibly to infections or other inflammatory diseases) may display blunted *I*_*Kr*_ with serious implications for cardiac repolarization especially in the setting of other known classical risk factors including electrolytes imbalance, QT_c_ prolonging medications, genetic and autoimmune channelopathies [22]. Our results predict specific inhibitors of IL-6R or cellular mediators that enhance channel opening to normalize *I*_*Kr*_ would be expected to correct QT_c_ prolongation in patients. By providing the first measurements of the effect of IL-6 on hERG/*I*_*Kr*_, we reveal that *I*_*Kr*_ is sensitive to pathological changes in cytokine levels in cardiac and systemic inflammation. Collectively our findings open new directions in determining the contribution of other downstream complex effectors (STAT3, MAPK, Pi3K/Akt) in IL-6-associated signaling pathways on *I*_*Kr*_. This study was undertaken in both native guinea-pig ventricular myocytes and HEK293 cells stably expressing hERG/*I*_*Kr*,_ both of which exhibited similar findings. It will be interesting to confirm these outcomes in other system models such as human induced pluripotent cells-derived cardiomyocytes or in vivo animal models. Regardless, the data clearly show that IL-6 depresses *I*_*Kr*_ through IL-6R and JAK pathways, suggesting a novel cytokine mechanism in acquired inflammation induced LQTS.

## Clinical perspectives

Pro-inflammatory IL-6 cytokine-mediated changes in cardiomyocyte ion channel function is a novel risk factor involved in the acquired inflammatory LQTS, a condition that underlies impaired repolarization, leading to ventricular arrhythmias and SCD. The translational implications are that patients with inflammatory disorders with high levels of IL-6 can benefit from routine ECG and counselling if other QT_c_ prolonging risk factors are present in these patients.
